# Successful Treatment With Rituximab for Severe Immune Thrombocytopenic Purpura During Hemodialysis

**DOI:** 10.7759/cureus.104592

**Published:** 2026-03-03

**Authors:** Mayuko Hanyuda, Yukihiro Wada, Tomomi Motohashi, Kiho Yanagisawa, Genichi Saito, Ryota Uchitsubo, Sayumi Kawamura,, Shun Sakurabayashi, Kazuhiro Takeuchi, Shokichi Naito, Yasuo Takeuchi

**Affiliations:** 1 Department of Nephrology, Kitasato University School of Medicine, Sagamihara, JPN

**Keywords:** bleeding risk, hemodialysis, immune thrombocytopenic purpura, myeloperoxidase-anti-neutrophil cytoplasmic antibody (mpo-anca), rituximab

## Abstract

Immune thrombocytopenic purpura (ITP), an autoimmune disorder characterized by peripheral platelet destruction, is uncommon in patients undergoing hemodialysis, who have an inherently elevated bleeding risk. We report a case of severe ITP in a 68-year-old woman on maintenance hemodialysis for 10 years due to myeloperoxidase-anti-neutrophil cytoplasmic antibody (MPO-ANCA)-associated glomerulonephritis, treated with rituximab. Three years before admission, she was diagnosed with rheumatoid arthritis (RA) and had unexplained thrombocytopenia that improved with prednisolone, resolving her arthritis. The disease activity of both MPO-ANCA-associated vasculitis and RA was later controlled without prednisolone. Four months prior, her platelet count suddenly dropped to 54,000/μL. Laboratory tests showed elevated platelet-associated IgG (PA-IgG), increased bone marrow megakaryocytes, negative antinuclear antibody, MPO-ANCA, anti-*Helicobacter pylori* antibody, and no splenomegaly, leading to a diagnosis of ITP. The absence of a temporal relationship with heparin exposure, the extremely low platelet count (<10,000/µL), and the preceding clinical course suggested that heparin-induced thrombocytopenia was unlikely. One month prior, the platelet count declined further to 3,000/μL; prednisolone (25 mg/day) was started without improvement. On admission, she had subcutaneous bleeding and an elevated immature platelet fraction. Eltrombopag was added but was ineffective. Rituximab (520 mg/body) was administered weekly for four weeks from day 9 after admission. By day 35, platelets improved to 175,000/μL with decreased immature platelet fraction and PA-IgG. Her condition stabilized, and prednisolone was tapered to 6 mg/day within four months post-discharge. Rituximab is a safe and effective treatment for severe ITP, even in hemodialysis patients, and is helpful for steadily tapering steroid therapy. According to a literature review of four ITP cases on dialysis, including our case, rituximab-based therapy is a reason.

## Introduction

Immune thrombocytopenic purpura (ITP) is an acquired autoimmune disorder characterized by immune-mediated platelet destruction and impaired megakaryopoiesis, resulting in thrombocytopenia despite normal bone marrow morphology [1]. ITP mainly affects primary hemostasis and typically presents with mucocutaneous bleeding, including purpura, epistaxis, ecchymoses, menorrhagia, and hematuria, although some patients may remain asymptomatic [2]. The incidence of ITP ranges from two to four cases per 100,000 person-years, with two age-related peaks: one between 20 and 30 years, showing a slight female predominance, and a larger one after 60 years, with an equal sex distribution [3]. ITP may occur as a primary condition or may be secondary to other disorders, such as exposure to certain medications; lymphoproliferative diseases, including Hodgkin’s lymphoma; infections (e.g., human immunodeficiency virus (HIV), hepatitis B virus (HBV), and hepatitis C virus (HCV), cytomegalovirus, Epstein-Barr virus (EBV), and *Helicobacter pylori*); autoimmune diseases such as systemic lupus erythematosus (SLE), rheumatoid arthritis (RA), and antiphospholipid syndrome (APS); and Evans syndrome [1].

The priorities of ITP treatment are to control active bleeding and to reduce the risk for future hemorrhagic events. Withdrawal of anticoagulant and antiplatelet agents, along with glucocorticoids and intravenous immune globulin (IVIG), constitutes the initial management of ITP [1]. Platelet transfusion is generally reserved for emergency situations involving severe or life-threatening bleeding [1]. Furthermore, in such serious or life-threatening situations, additional therapeutic interventions may be required. For patients who fail to achieve an initial response to glucocorticoids or who experience recurrent declines in platelet counts after tapering glucocorticoids, other medical therapies such as thrombopoietin-receptor agonists (e.g., eltrombopag: EPAG) and immunomodulatory agents like rituximab are considered [1]. To date, the safety and efficacy of rituximab, an anti-CD20 antibody, in adult patients with ITP have been demonstrated in multiple clinical studies [4-7].

Herein, we present a case of ITP complicated with autoimmune diseases in a patient undergoing hemodialysis, in whom intensive therapy with rituximab proved effective. To our knowledge, there have been relatively few reports of hemodialysis patients with ITP treated with rituximab, and the efficacy and safety of rituximab in this population have not been sufficiently established. In this article, we review four previously reported cases of dialysis patients with ITP, along with the present case, to highlight the rarity and clinical significance of this condition.

## Case presentation

A 68-year-old woman was referred to our hospital due to subcutaneous bleeding of the extremities. Ten years earlier, she had initiated maintenance hemodialysis for end-stage renal disease caused by myeloperoxidase-antineutrophil cytoplasmic antibody (MPO-ANCA)-associated vasculitis (microscopic polyangiitis, mPA). Because of recurrent vascular access occlusion unrelated to the use of heparin as an anticoagulant, a permanent tunneled hemodialysis catheter had been required. Three years previously, the patient developed RA and presented with unexplained thrombocytopenia; however, both thrombocytopenia and arthritis improved with corticosteroid therapy for RA. Thereafter, the disease activity of both mPA and RA remained stable without corticosteroid treatment. Four months before admission, her platelet count suddenly decreased to 54,000/µL. Laboratory tests revealed elevated platelet-associated IgG (PA-IgG) levels of 999 EU. Bone marrow examination revealed hypercellularity with increased megakaryocytes. Tests for anti-*H. pylori* antibodies, splenomegaly, fever, and arthralgia were all negative. Rheumatoid factor (RF) was positive, whereas antinuclear antibodies (ANAs) and MPO-ANCA were negative. Based on these findings, the referring physician diagnosed idiopathic ITP at that time. One month prior to transfer, her platelet count further declined to 3,000/µL. She received dexamethasone 20 mg/day for three days, followed by prednisolone (PSL) 25 mg/day, but no improvement was observed. EPAG at 12.5 mg/day was initiated; however, the thrombocytopenia remained refractory. The patient was therefore transferred to our hospital for further evaluation and management, and concurrent hemodialysis care was continued in our department.

On admission, body temperature was 36.5℃, heart rate was 57 beats/min, and blood pressure was 105/56 mmHg. Her height and dry weight are 153 cm and 45 kg, respectively, and her body surface area was calculated to be 1.38 m². No abnormalities were observed in the chest or abdomen, and no edema was present. There was no cervical lymphadenopathy or joint swelling; however, scattered subcutaneous hemorrhages were noted on the extremities. The principal laboratory findings are presented in Table 1. A complete blood count revealed mild anemia and marked thrombocytopenia (platelet count, 0.3 × 10⁴/mm³), with a markedly elevated immature platelet fraction (IPF) of 36.5%. Coagulation studies were unremarkable. Serum biochemistry showed no evidence of hypoproteinemia, liver dysfunction, or electrolyte imbalance; however, severe renal dysfunction was present. Immunological studies revealed no elevation of C-reactive protein, hypocomplementemia, or hypergammaglobulinemia, whereas elevated levels of matrix metalloproteinase-3 (MMP-3) (204.7 ng/mL) were detected. Furthermore, RF (262 IU/mL) and anti-cardiolipin IgG antibody (24.2 U/mL) were positive. Negative results were obtained for anti-nuclear antibody (ANA), anti-double-stranded-DNA antibody, MPO-ANCA, and proteinase 3-ANCA. Serum hepatitis B surface antigen and anti-hepatitis C virus antibody findings were also negative. Collectively, her clinical course and laboratory findings supported the diagnosis of ITP complicated with inactive mPA and RA.

**Table 1:** Principal laboratory findings on admission Abbreviations: WBC, white blood cell; Hb, hemoglobin; Plt, platelet; IPF, immature platelet fraction; PT-T, prothrombin time; Sec, second; APTT, activated partial thromboplastin time, Fib, fibrinogen; SF, soluble fibrin; PIC, plasmin-alpha2-plasmin inhibitor-complex; TP, total protein; Alb, albumin; T-bil, total bilirubin; GOT, aspartate aminotransferase; GPT, alanine aminotransferase; LDH, lactate dehydrogenase; BUN, blood urea nitrogen; Cr, creatinine; Na, sodium; K, potassium; Cl, chloride; Ca, calcium; P, phosphorus; CRP, C-reactive protein; IgG, immunoglobulin G; IgA, Imunoglobulin A; IgM, immunoglobulin M; C3, component 3; C4, component 4; CH50, total hemolytic complement activity; ANA, antinuclear antibody; Anti-double stranded-DNA Ab; RF, rheumatoid Factor; MMP3, matrix metalloproteinase-3; MPO-ANCA, myeloperoxidase antineutrophil cytoplasmic antibody; PR3-ANCA, proteinase 3 antineutrophil cytoplasmic antibody; Anti-CL Ab, anti-cardiolipin IgG antibody; PA-IgG, platelet-associated IgG

Parameter	Unit	Value	Reference range
Hematology			
WBC count	µL^-1^	6,000	3,300-8,600
Hemoglobin	g/dL	10.5	13.7-16.8
Platelet count	10^4^/µL	0.3	15.8-34.8
IPF	%	36.5	1.1-6.1
Coagulation			
PT-T	sec	12.8	10.0-13.0
APTT	sec	25.9	23.0-38.0
Fib	mg/dL	126	200-400
D-dimer	μg/mL	1.2	0.0-1.0
SF	μg/mL	9.7	<5.0
PIC	μg/mL	1.0	<0.8
Blood chemistry			
TP	g/dL	6.2	6.0-8.0
Alb	g/dL	3.6	4.1-5.1
T-bil	mg/dL	0.3	0.2-1.2
GOT	IU/L	10	10-40
GPT	IU/L	6	10-40
LDH	IU/L	324	140-280
BUN	mg/dL	44.9	8.0-20.0
Cr	mg/dL	5.9	0.6-1.0
Na	mEq/L	136	138-145
K	mEq/L	3.4	3.5-5.0
Cl	mEq/L	102	101-108
Ca	mg/dL	8.5	8.8-10.0
P	mg/dL	3.2	2.5-4.7
Immunological test			
CRP	mg/dL	0.05	0.0-0.3
Ferritin	ng/dL	38	15-150
IgG	mg/dL	1310	870-1700
IgA	mg/dL	477	110-410
IgM	mg/dL	77	52-270
C3	mg/dL	86	80-140
C4	mg/dL	32	15-45
CH50	U/mL	43	30-45
ANA	titer	negative	
Anti-ds-DNA Ab	IU/mL	negative	
RF	IU/mL	262.0	0-20.0
MMP-3	ng/mL	204.7	17.3-59.7
MPO-ANCA	IU/mL	negative	
PR3-ANCA	IU/mL	negative	
Anti-CL Ab	U/mL	24.2	<12.3
PA-IgG	ng/10^7^ cells	999	<30.2

**Figure 1:**
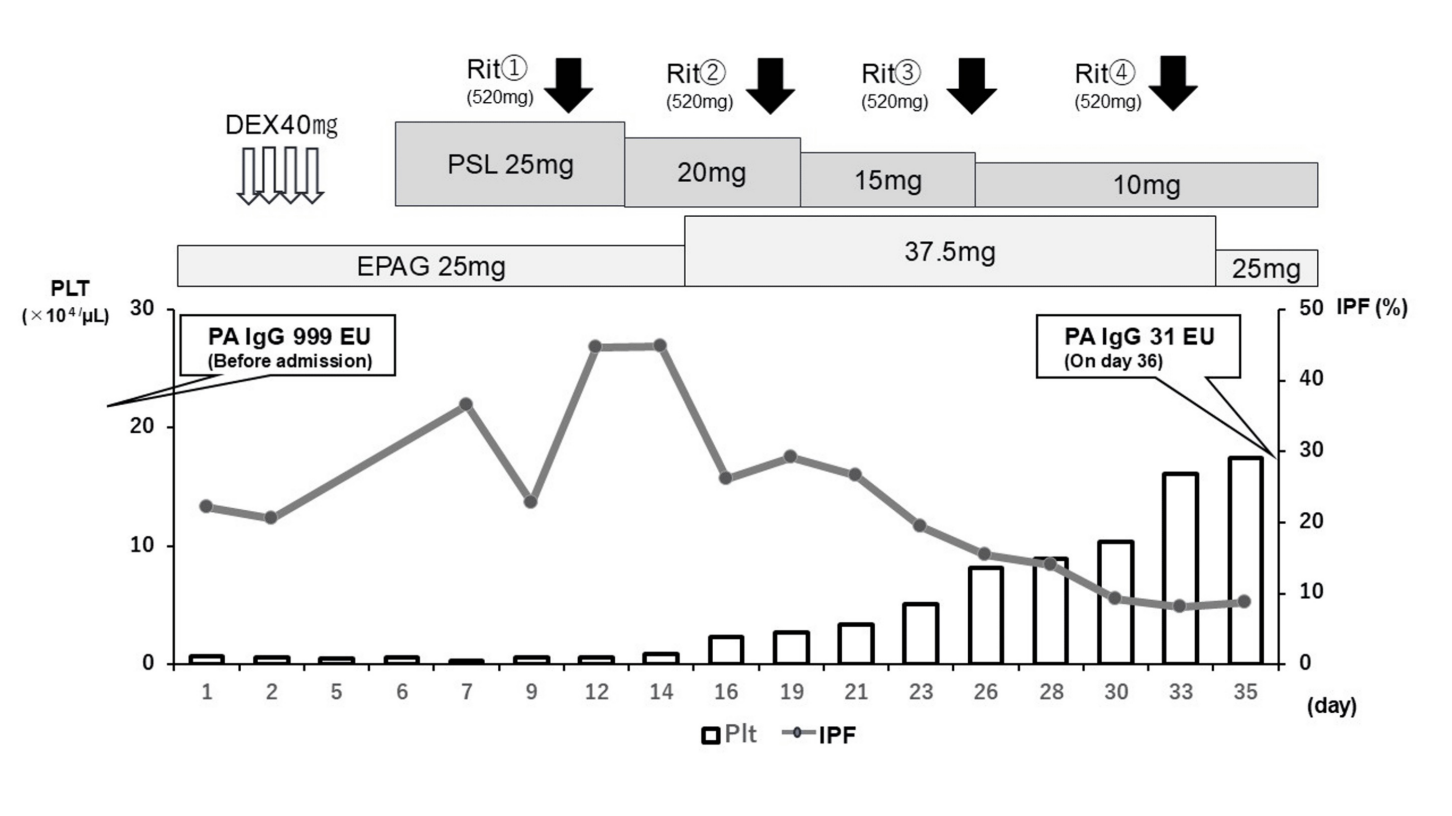
Clinical course and treatment of the patient after admission. Abbreviations: DEX, dexamethasone; Rit, rituximab; PSL, prednisolone; EPAG, eltrombopag; PLT, platelet; IPF, immature platelet fraction; PA-IgG, platelet-associated IgG

The clinical course after admission is summarized in Figure 1. Initially, dexamethasone was administered for four days while EPAG and PSL were continued. There was no worsening of serious bleeding, and hemostasis was adequately maintained while minimizing the potential thrombotic risk from platelet supplementation. Thereafter, the dose of EPAG was increased to 25 mg/day; however, thrombocytopenia persisted. Therefore, from hospital day 9 after admission, rituximab (520 mg/body) was administered weekly for four consecutive weeks. Consequently, the platelet gradually increased to 175,000/µL, with the reduction of IPF to 8% and PA-IgG to 31 EU, respectively, on day 35 after admission. No thrombotic events were observed during the clinical course. No coagulation was noted on the dialyzer during hemodialysis sessions. Hemodialysis was performed via a tunneled catheter without any bleeding complications or thrombosis. Ultimately, her condition stabilized, and PSL was rapidly tapered to 6 mg/day within four months post-discharge. Throughout the clinical course, low-molecular-weight heparin (initial bolus of 500 units followed by continuous infusion of 500 units) was used for anticoagulation during hemodialysis, as prescribed at the referring hospital. The anticoagulation regimen was not modified during either the period of the lowest platelet count or the recovery phase. No thrombotic events or dialysis circuit clotting were observed. Although the anti-cardiolipin IgG antibody was positive, repeat testing at ≥12 weeks was not performed; therefore, the possibility of APS cannot be excluded. However, no thrombosis occurred in the long-term tunneled dialysis catheter, and the dialysis circuits remained free of clotting throughout the clinical course.

## Discussion

Based on the diagnostic criteria for ITP [1], the present case was highly compatible with ITP because of the marked thrombocytopenia accompanied by elevated IPF in peripheral blood, the positivity of PA-IgG, and increased megakaryocytes in the bone marrow. Other potential causes of thrombocytopenia, including chronic liver disease, acute leukemia, aplastic anemia, and genetic disorders such as Bernard-Soulier syndrome or MYH9-related disorders, as well as thrombotic thrombocytopenic purpura, could be ruled out. Regarding the possibility of heparin-induced thrombocytopenia (HIT), neither the platelet factor 4-heparin antibody test nor platelet-activation assays could be assessed. However, the recurrent vascular access occlusions observed previously were unrelated to heparin use. Moreover, previous reports indicated that platelet counts <10,000/µL, as observed in this case, were uncommon among hemodialysis patients with HIT [8,9], making the diagnosis less likely. As for the etiology of ITP, secondary autoimmune disease-related ITP could not be entirely ruled out. Although ANA, ANCA, and anti-DNA antibodies were negative and the activity of mPA was inactive at the onset of ITP, and no venous thrombosis was observed during the clinical course, the patient demonstrated elevated MMP-3 levels and positivity for RF and anti-cardiolipin IgG antibody detected in APS. Taken together, these findings suggest that the ITP in the present case may have developed as a secondary autoimmune condition rather than primary ITP. The patient was also considered to have severe ITP refractory to conventional therapy.

We conducted a comprehensive literature search from 1990 through December 2025 using PubMed and relevant Japanese databases, screening not only abstracts but also full texts of case reports. Based on this search, we identified only three published cases of hemodialysis patients with ITP [10-12]. Table 2 summarizes the clinical characteristics of these previously reported cases, together with the present case. All patients were female and aged 50-75 years. The etiology of ITP varied among cases: two were secondary to autoimmune diseases, one was secondary to COVID-19 infection, and one represented primary ITP. Initial platelet counts were markedly reduced in all cases; notably, three of the four patients had platelet counts below 10,000/µL. When available, PA-IgG levels were elevated. Of note, all cases demonstrated resistance to standard first-line therapy, which typically included PSL in combination with IVIG or EPAG [1]. Rituximab was administered in three of the four cases, including the present case. In case 2, a patient with primary ITP on dialysis showed a favorable clinical course with danazol without rituximab [11]. In general, hemodialysis patients have an intrinsically increased risk of bleeding [13,14]. Arteriovenous fistula (AVF) access and repeated exposure to anti-coagulant agents such as heparin during dialysis are considered to be significant contributors to this higher bleeding risk compared with the general population [13,14]. According to a recent review, the rate of major bleeding events among dialysis patients ranges from 25 to 80 per 1,000 person-years, whereas the corresponding rate in the general population is only 0.5 to 0.9 per 1,000 person-years [13]. In the present case, hemodialysis was performed using a permanent tunneled catheter, not an AVF, which may have been advantageous in preventing catastrophic bleeding in the setting of profound thrombocytopenia. In line with the previously reported cases summarized in Table 2, we suggest that early and intensive treatment for therapy-resistant ITP might be essential to avoid severe clinical deterioration in patients undergoing hemodialysis.

**Table 2:** Clinical features of previously reported cases of ITP in hemodialysis patients Abbreviations: ITP, immune thrombocytopenic purpura; Ref, reference; yr, year; Plt, platelet count; PA-IgG, platelet-associated immunoglobulin G; EU, enzyme-linked immunosorbent assay units; Rit, rituximab; F, female; NA, not available; PSL, prednisolone; IVIG, intravenous immunoglobulin; EPAG, eltrombopag; CyA, cyclosporin A; CR, complete remission; SLE, systemic lupus erythematosus; mPA, microscopic polyangiitis; RA, rheumatoid arthritis

Case	Etiology [Reference]	Age (year)/sex	Plt (/mL)	PA-IgG (EU)	Clinical course/treatment	Rit	Outcome
1	Secondary (COVID-19) [10]	75/F	5,000	NA	Therapy resistance/ PSL + IVIG + EPAG + Rit	yes	CR
2	Primary [11]	71/F	4,000	385.4	Therapy resistance/ PSL + EPAG + danazol	no	CR
3	Secondary (SLE) [12]	50/F	18,000	NA	Therapy resistance/ PSL + Rit	yes	NA
4	Secondary (mPA+RA) [This case]	68/F	3,000	999	Therapy resistance/ PSL + EPAG + Rit	yes	CR

In Japan, rituximab has been administered as a second-line therapy for ITP in patients who are unresponsive or intolerant to corticosteroids [6]. Rituximab is an anti-CD20 monoclonal antibody that induces apoptosis of CD20-expressing lymphocytes through antibody-dependent cell-mediated cytotoxicity and complement-mediated lysis [7]. Because ITP is fundamentally caused by antiplatelet autoantibodies, leading both to accelerated platelet destruction in the reticuloendothelial system and to impaired platelet production via inhibition of megakaryocyte growth, B cells play a central role in its pathophysiology. Accordingly, B-cell depletion with rituximab represents a rational and mechanistically appropriate therapeutic strategy for ITP. Moreover, rituximab is considered to be helpful for the gradual and successful tapering of steroid therapy. Given the risk of opportunistic infections in hemodialysis patients, the ability to taper steroids more rapidly might be extremely valuable for patient management. Rituximab is now used worldwide, not only in Japan, and is internationally accepted as a second-line therapy for refractory ITP [1,5,7,15,16]. A systematic review reported an overall response rate of 60% and a complete response (CR) rate of 40% among patients with ITP [16]. Moreover, a meta-analysis of five randomized controlled trials demonstrated a significantly higher incidence of CR at 6 months with rituximab than with glucocorticoids or placebo [17]. Furthermore, rituximab provides sustained platelet responses, with approximately 50% of responders maintaining adequate platelet counts for more than 2 years [18,19]. In a clinical setting, rituximab is usually administered at 375 mg/m² once weekly for four consecutive weeks, regardless of renal function or the presence of renal impairment [1,5], as in the present case. Given the widespread use of rituximab in managing other disorders, including mPA and nephrotic syndrome [20], it is considered a well-established treatment in nephrology. Regarding the evidence for the efficacy of rituximab in hemodialysis patients with ITP, only a few cases have been reported, as summarized in Table 1. Among the reported hemodialysis patients who received rituximab, two achieved complete remission, suggesting that rituximab may offer therapeutic benefit in this population despite their inherent bleeding risk. In addition, new therapeutic options, including the Syk inhibitor fostamatinib and the FcRn inhibitor efgartigimod, have recently been approved as second-line treatments for ITP in Japan [6]. Several other novel agents, such as Bruton tyrosine kinase inhibitors, BAFF-receptor inhibitors, and anti-CD38 antibodies, are currently under development [7]. In short, the above-described targeted therapies, in addition to rituximab, have increasingly been used as second-line treatments for ITP in non-dialysis patients. However, in the case of dialysis patients with multiple comorbidities, the administration of these novel agents needs to be supported by sufficient clinical evidence to ensure their safety. Meanwhile, the present review suggests that the safety of rituximab might be relatively assured in hemodialysis patients with ITP. Thus, first and foremost, further accumulation and analysis of cases of ITP in hemodialysis patients are required to assess the efficacy of rituximab.

## Conclusions

Rituximab might represent an effective and feasible therapeutic option for refractory ITP in patients undergoing hemodialysis, a population at particularly high risk of bleeding and therapeutic complications. In the present case, rituximab induced a sustained platelet response without dialysis circuit clotting, thrombotic events, or infectious complications, and enabled successful tapering of corticosteroid therapy. These findings are clinically meaningful because hemodialysis patients are intrinsically predisposed to both bleeding and infection, making prolonged high-dose corticosteroid therapy particularly problematic. Although the number of reported cases remains limited, our case adds to the growing evidence that rituximab can be administered safely in this setting. Further accumulation of cases and systematic investigations are warranted to establish optimal treatment strategies and to better define the risk-benefit profile of rituximab in dialysis-dependent patients with ITP.
